# Operation for Pharaphimosis

**Published:** 1856-08

**Authors:** 


*Operation for Pharaphimosis.*—At the session of the Academy of Sciences (Paris), on the 21st of April, the following extract was read from a letter of M. Malgaigne:—

"With this strangulation, as with strangulated hernia, we attempt at first to accomplish reduction, and usually succeed. But when reduction is impossible, it is advised, as with strangulated hernia, to divide the bridle which strangulates it, even if it is necessary to repeat this section at two or three points. The danger of strangulation is thus diminished, but the reduction still continues to be impossible. At least I have never seen it accomplished after such an operation. What is the reason of this want of success? It is that the preputial ring, in producing inflammation, ulceration, sometimes even gangrene, of the parts strangulated, commences by thickening the subjacent cellular tissue, and by producing extensive adhesions between the integument and the cavernous bodies. Dividing the stricture, though repeated, does not destroy these adhesions, and does not suffice for the reduction, while destruction of these adhesions, even without division of the stricture, is sufficient to allow the return of the parts to their place.

"Thus the study of this affection has led me to distinguish a new element, hitherto left in the shade. The establishment of this element gave a new indication, and this is the way in which I have met this indication.

"A young man came under my care the 11th of this month, for a paraphimosis of five days' duration, and already there was seen upon the back of the penis a superficial ulceration, embracing more than half of the circumference of the organ. The *internes* tried to reduce it without success. The next day, at the visit, I was no more fortunate; the adhesions of the integument to the cancerous bodies presented an insurmountable obstacle to it. I slipped a narrow bistoury flatways between the integuments and the cavernous bodies, by means of which I divided those adhesions to the extent of one centimetre (four-tenths of an inch). This did not suffice. I carried a probe-pointed bistoury into the incision, to complete the division of the adhesions throughout their whole extent, and the reduction was accomplished with the greatest facility. The next day, the engorgement of the prepuce had diminished, the third day the ulcerated surface had cicatrized, and the patient went out the 20th of April, having been well several days, and without experiencing any kind of accident."—*Am. Med. Monthly.*

